# Team-Based Care for Improving Hypertension Management: A Pragmatic Randomized Controlled Trial

**DOI:** 10.3389/fcvm.2021.760662

**Published:** 2021-10-25

**Authors:** Valérie Santschi, Gregoire Wuerzner, Bruno Pais, Arnaud Chiolero, Philippe Schaller, Lyne Cloutier, Gilles Paradis, Michel Burnier

**Affiliations:** ^1^La Source, School of Nursing Sciences, HES-SO University of Applied Sciences and Arts Western Switzerland, Lausanne, Switzerland; ^2^Service of Nephrology and Hypertension, Lausanne University Hospital, University of Lausanne, Lausanne, Switzerland; ^3^Population Health Laboratory, #PopHealthLab, University of Fribourg, Fribourg, Switzerland; ^4^Institute of Primary Health Care (BIHAM), University of Bern, Bern, Switzerland; ^5^School of Population and Global Health, McGill University, Montreal, QC, Canada; ^6^Cité Générations, Onex, Switzerland; ^7^Département des sciences infirmières, Université du Québec à Trois-Rivières, Trois-Rivières, QC, Canada

**Keywords:** hypertension, team-based care, healthcare professionals, healthcare services research, interprofessional intervention

## Abstract

**Objective:** We evaluated the effect on long term blood pressure (BP) of an interprofessional team-based care (TBC) intervention, involving nurses, pharmacists, and physicians, compared to usual care.

**Methods:** We conducted a pragmatic randomized controlled study in ambulatory clinics and community pharmacies in Switzerland (ClinicalTrials.gov: NCT02511093). Uncontrolled treated hypertensive patients were randomized to TBC or usual care (UC). In the TBC group, nurses and pharmacists met patients every 6 weeks to measure BP, assess lifestyle, support medication adherence, and provide health education for 6 months. After each visit, they wrote a report to the physician who could adjust antihypertensive therapy. The outcome was the intention-to-treat difference in mean daytime ambulatory blood pressure measurement (ABPM) and control (<135/85 mmHg) at 6 and 12 months.

**Results:** Eighty-nine patients (60 men/29 women; mean (SD) age: 61(12) year) were randomized to TBC (*n* = 43) or UC (*n* = 46). At baseline, mean (SD) BP was 144(10)/90(8) mmHg and 147(12)/87(11) mmHg in the TBC and UC groups. At 6 months, the between-groups difference in daytime systolic ABPM was−3 mmHg [95% confidence interval (CI):−10 to +4; *p* = 0.45]; at 12 months, this difference was−7 mmHg [95% CI:−13 to−2; *p* = 0.01]. At 6 months, the between-groups difference in daytime diastolic ABPM was +2 mmHg [95% CI:−1 to +6; *p* = 0.20]; at 12 months, this difference was−2 mmHg [95% CI:−5 to +2; 0.42]. Upon adjustment for baseline covariates including baseline BP, the between-groups differences at 6 and 12 months were maintained. At 6 months, there was no difference in BP control. At 12 months, the TBC group tended to have a better control in systolic BP (*p* = 0.07) but not in diastolic BP (*p* = 0.33).

**Conclusion:** While there was not significant effect on BP at 6 months of follow-up, the TBC intervention can help decrease long-term systolic BP among uncontrolled hypertensive patients.

## Introduction

Hypertension is a major risk factor for stroke and cardiovascular diseases and a major cause of mortality worldwide (1). One quarter to one third of European adults have hypertension, and this burden will increase due to the aging of the population (2). Despite effective blood pressure (BP) lowering drugs to prevent cardiovascular events and reduce mortality (3), a large proportion of patients with hypertension remain uncontrolled (4–6). In responses to these challenges, innovative models of care are needed to improve BP control, such as team-based care (TBC) approaches that include pharmacists and nurses in primary care (7, 8).

Various studies involving pharmacists or nurses in primary care have shown that they can help improve BP control (9–13). Moreover, the evidence from systematic reviews with meta-analysis supports that pharmacists—working alone or in teams (8, 14)—can improve the management of hypertension as well other cardiovascular risk factors (15, 16). Another systematic review found evidence that nurses led interventions are effective in the management of BP (17). Since 2014, the U.S Community Preventive Services Task Force recommends TBC for hypertension management (7, 18). TBC is defined as a coordinated model of care involving different healthcare professionals, such as physicians and other non-physician clinicians such as pharmacists, nurses, working in collaborative partnership, each with their own expertise, to manage hypertension, and optimize patient education. Recent guidelines on hypertension management, notably the 2017 guidelines from the American College of Cardiology and the American Heart Association (ACC/AHA) as well as the 2018 guidelines of the European Society of Cardiology and the European Society of Hypertension (ESC/ESH) recommend TBC for the first time with the involvement of pharmacists and nurses in the management of hypertension (19–21).

However, high quality evidence showing the efficacy of TBC in hypertension comes essentially from randomized controlled studies conducted in North America in outpatient clinics or by general practitioners and with a median duration of follow-up of 6 month (8). The TBC model to improve long term BP with both nurses and pharmacists need therefore to be evaluated in a European real-life primary care setting. The objective of the TBC for improving Hypertension management (TBC-HTA) randomized controlled study was to assess whether a TBC intervention, involving nurses and community pharmacists working in collaboration with physicians, improves long term daytime ambulatory BP among uncontrolled treated hypertensive patients in primary care practices under real-life conditions (22).

## Materials and Methods

### Study Design, Setting and Participants

This study was a pragmatic randomized controlled trial conducted from September 2014 to December 2019 comparing a 6-month TBC interprofessional intervention among outpatients followed in ambulatory clinics and community pharmacies in Lausanne and Geneva, Switzerland (ClinicalTrials.gov: NCT02511093), which were included in the study on a voluntary basis. The details of the study protocol have been published previously (22). The ethics committees of the cantons of Vaud (CER-VD 449/13) and Geneva (CCER GE 15/281), Switzerland approved the study protocol which followed the principles of the Declaration of Helsinki.

Outpatient clinic databases built on the electronic medical records of patients followed-up in the ambulatory clinics, were used to identify patients. To be selected, patients had the following inclusion criteria: (1) uncontrolled hypertension defined as daytime ambulatory BP ≥ 135/85 mmHg or office BP ≥ 140/90 mmHg over at least two consecutive visits; (2) taking at least one antihypertensive medication; (3) aged 18 years old or more; (4) speak and understand French; (5) agree to use the service from the same pharmacy for the duration of the study. Exclusion criteria were (1) pregnancy or lactation, (2) hospitalization, (3) living in a nursing home, (4) inability to understand the study aim, (5) participation in another clinical study, or (6) daytime ambulatory BP > 180/110 mmHg.

Patients meeting inclusion criteria were approached during a routine clinic visit by the physician or contacted by phone by a nurse who explained the study and ascertained patients' willingness to participate. If patients agreed to participate, and provide a written consent form, a research clinic visit was scheduled at the ambulatory clinic. Demographic data were collected at baseline, including sex, age, comorbid conditions, and the number and type of antihypertensive drugs. After completing the baseline assessment, patients were randomized *via* a computer number generated using sequentially numbered opaque sealed envelopes in an equal allocation ratio (1:1) using permuted blocks to either the 6-month TBC intervention group or the UC care group (22). Due to the nature of the intervention, patients and healthcare professionals (physicians, nurses and community pharmacists) could not be blinded to the allocation.

### TBC Intervention

Each patient allocated to the TBC group received the TBC interprofessional intervention from nurses and community pharmacists working in collaboration with physicians. Prior to the study, nurses and community pharmacists were trained during a 2-h workshop about the study requirements, TBC intervention, standardized BP measurement and hypertension care according to the ESC/ESH recommendations (22), antihypertensive medication management, and counseling about lifestyle modification (physical activity and diet). More precisely, the TBC intervention, based on specific competencies of healthcare professionals, comprises (22):

1. A structured individual intervention conducted by trained nurses and community pharmacists every 6 weeks (at baseline, 6-, 12-, 18-week) during the 6-month of follow-up.
2. At each visit, patients received structured individual interventions conducted by trained nurses and community pharmacists (BP measurement, assessment and counseling about lifestyle and medication adherence and, health education concerning treatment and disease), respectively.
3. Following each 6-week visit, a summary report (BP measures, medication adherence and lifestyle assessment) with recommendations were prepared by the nurse and the pharmacist for the physician who adjusted antihypertensive therapy accordingly.

No medication change was allowed during the first 6 weeks of follow-up. If BP was uncontrolled (≥140/90 mmHg) at the 6, 12, and 18-week sessions with the community pharmacist or the nurse, the physician was informed by phone or in face-to-face meeting. The physician then adapted the treatment as needed taking account the nurse's and the community pharmacist's recommendations on lifestyle, medication adherence, and therapy.

### Usual Care

Patients allocated to UC group received routine care by their habitual physician without any specific nurse or community pharmacist intervention. They attended schedule visits at baseline, 6 and 12 months of follow-up, where ABPM was taken.

### Blood Pressure Measurement

At baseline, 6- and 12-months (i.e., 6 months post-intervention), daytime ABPM, used as the main outcome, was taken in TBC and UC patients using clinically validated electronic devices, and using a standardized protocol (22) in line with the European Society of Hypertension (ESH) recommendations (23). More precisely, the ABPM device was installed on the dominant arm by the nurse who explains the procedure to the patient. Measurements were based on the auscultatory mode, relayed by the oscillometric mode in case of failure of the auscultatory mode. Measurements were made every 20-min interval during the day and every 60-min interval during the night (23). The device used was the electronic Diasys (DIASYS integra; Novacor SA, Rueil-Malmaison, France) or Boso (Bosch+Sohn, Allemagne).

If ABPM was not available at baseline, BP was based on automated office BP measurements and computed as the average of the last 3 out of 6 measurements with the patient resting alone quietly (24). In the TBC group, every 6 weeks, automated office BP was measured by the nurse and by the community pharmacist using the Microlife WatchBP home, a clinically validated oscillometric device (25).

### Outcome

The primary outcome was the difference in mean systolic/diastolic daytime ABPM between TBC and UC patients and the difference in the proportion of TBC and UC patients with controlled systolic/diastolic daytime ABPM (<135/85 mmHg) at 6-month. The secondary outcome was the difference in mean systolic/diastolic daytime ABPM between TBC and UC patients and the difference in the proportion of TBC and UC patients with controlled systolic/diastolic daytime ABP (<135/85 mmHg) at 12-month (6 months post-intervention).

Other outcomes included the number, classes and daily dosages of antihypertensive drugs taken during the study that were documented using medical electronic records at 6- and 12-month (6 months post-intervention follow-up). The differences in mean number, modification, intensification and reduction of antihypertensive drugs were also assessed. Using the start and end dates of prescribed drugs, we defined antihypertensive-drug modifications as drug changes (changing one class of drug for another), or drug intensifications (adding a new drug or increasing a drug dosage), or drug reductions (stopping a drug without replacing it or decreasing a drug dosage) (26).

### Sample Size and Statistical Analysis

Based on the results of the systematic review by Santschi et al. assessing the impact of pharmacist interventions on BP, a difference in systolic BP of 6 to 10 mmHg was expected between TBC and UC groups. A sample size of 46 patients per group provided 80% power to detect a 6 mmHg difference in systolic BP (SD: 10 mmHg) with a two-sided alpha of 5%. Assuming a drop-out or loss to follow-up rate of ~15%, the targeted sample size was adjusted to 55 per group, for a total sample size of 110.

Descriptive statistics were used to present baseline characteristics of TBC and UC patients as number, percentage and means (standard deviation). For the per-protocol analysis, missing BP value at 6 or 12 months were not imputed; the analyses were conducted on patients with complete follow-up and no missing BP data. For the ITT analyses, the last observation carried forward method was used for missing data (27). We imputed missing values for measurements at 6 months of follow-up (TBC: 4 patients; UC: 4 patients) or at 12 months of follow-up (TBC: 5 patients; UC: 8 patients) of follow-up. As planned, main analyses were followed the intention-to-treat (ITT) principle (22) and used Student's two-sided *t*-test to assess the statistical significance of the ITT between-groups difference in systolic and diastolic ambulatory BP at 6- and 12-month of follow-up. The statistical significance for the ITT between-groups difference in the proportion of patients with systolic/diastolic BP control were calculated at 6- and 12-month of follow-up using a chi-squared test. Further, in addition of the main analyses and following recent recommendations for the analyses of pragmatic randomized trials (28), along first the ITT principle and second the per-protocol principle, a set of linear regression analyses were conducted to account for the potential biasing effect of differences in baseline characteristics, especially baseline BP level, between the TBC and the UC groups on the outcomes. Hence, to assess the association of group allocation with systolic and diastolic daytime BP, respectively, three regression models of growing complexity were fitted with (1) no adjustment; (2) adjustments for age, sex, and recruitment center and (3) additional adjustments for the number of antihypertensive treatment at baseline and for BP at baseline (29). Two-sided *P*-value <0.05 was considered as statistically significant. All statistical analyses were conducted with Stata software version 16.0 (Stata Corp, College Station, TX, USA) and Microsoft Excel (version 16).

## Results

**Figure 1:**
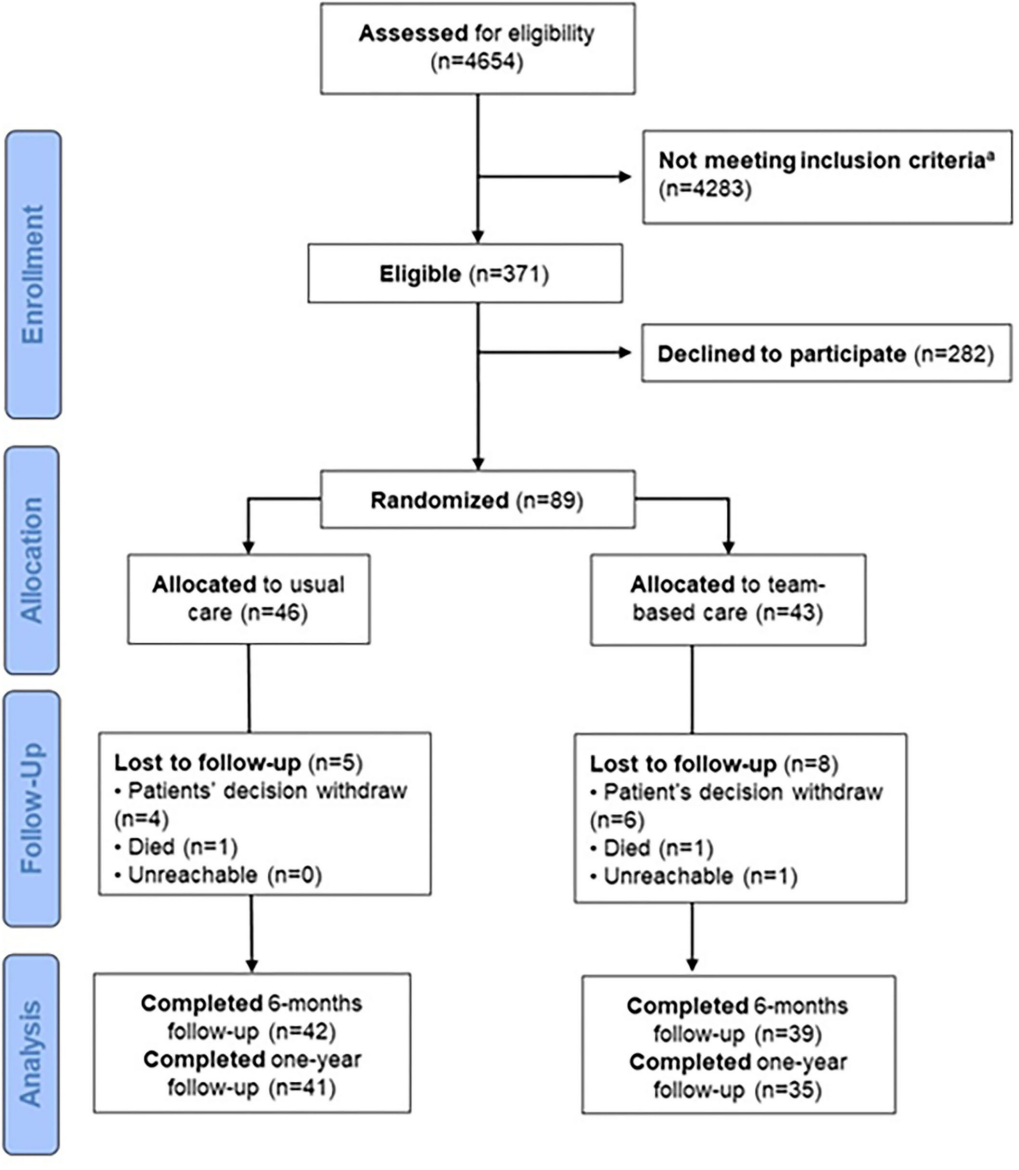
Flow diagram of included patients. ^*a*^Of the 4,283 who did not meet inclusion criteria, 195 have HTN drug treatment since <1 months, 1,388 did not have HTN drug treatment, 1,063 did not have recent BP measurement, 315 were aged <18 years, 486 were hospitalized or nursing home and 320 were patients not followed by participating physicians.

In total, 4,654 patients were assessed for eligibility using the ambulatory clinic database and 371 were identified as potentially eligible (Figure 1). As underlined in the Figure 1, the number of participants who did not meet all inclusion criteria or had exclusion criteria—i.e., no hypertension drug treatment, no recent BP measurement, aged <18 years old, hospitalized, living in nursing home or not followed-up by a participating physician—were documented. Eighty-nine patients (24% of potentially eligible) agreed to participate and were included in the study: 43 patients were randomly assigned to TBC and 46 patients to UC group. Of these, 81 (91%) (TBC: 39; UC: 42) completed the 6-month of follow-up, and 76 (85%) (TBC: 35; UC: 41) completed the 12-month follow-up.

Table 1 summarizes the baseline characteristics of the 89 included patients. The mean age was 60 (SD: 12) years and two thirds of patients were men. More than 50% of patients were obese and 12% were current smokers. Patients took on average 3 (SD: 2 drugs) (to treat hypertension and other conditions) daily, and more than 50% were treated with 4 drugs or more per day. A large proportion of patients had comorbidities, such as cardiovascular diseases, diabetes, dyslipidaemia, and chronic kidney disease. In the TBC group, systolic BP was slightly lower and diastolic BP slightly higher compared to the UC group. To account for a potential biasing effect of these differences on the outcomes, regression analyses adjusted for baseline BP were conducted (see below).

**Table 1:** Baseline characteristics of the patients.

	**UC**	**TBC**
Patients, *n*	46	43
Sex, (men/women), *n*	29/17	31/12
Mean age, years (SD)	61 (13)	60 (11)
Current smoker, *n* (%)	6 (13%)	5 (12%)
Mean BMI, kg/m^2^ (SD)	28.0 (4.6)	30.6 (6.5)
**Comorbid conditions**		
Cardiovascular disease, *n* (%)	9 (20%)	9 (21%)
Diabetes mellitus, *n* (%)	7 (15%)	14 (33%)
Dyslipidemia, *n* (%)	19 (41%)	17 (40%)
Chronic kidney disease, *n* (%)	3 (7%)	4 (9%)
Mean number of all prescription drugs, *n* (SD) [min; max]	3.0 (1.8) [1; 9]	3.2 (2.0) [1; 10]
Polymedication (3 drugs or more), *n* (%)	25 (54%)	25 (58%)
Mean time since hypertension diagnosis, years (SD) [min; max]	7.5 (7.9) [0; 30]	11.7 (11.7) [0; 35]

*UC, usual care; TBC, team-based care; SD, standard deviation; BMI, body mass index; smoking, current smoking ≥1 cigarette/day*.

### Blood Pressure and Antihypertensive Treatment

Table 2 summarizes information about baseline BP and antihypertensive treatment. TBC and UC patients were treated most often with angiotensin receptor blockers (55%), diuretics (44%), angiotensin converting enzyme inhibitors (30%), calcium channel blockers (30%), and beta-blockers (22%). The mean number of daily antihypertensive drugs taken was 2 (SD: 1; range: 1–4) in both groups, with 38% of patients taking one drug per day, 40% two drugs per day, and 22% three drugs or more per day. No major clinical difference was observed between TBC and UC groups regarding the baseline BP and treatment for hypertension.

**Table 2:** Baseline blood pressure (BP) and treatment for hypertension.

	**UC**	**TBC**
	***n* = 46**	***n* = 43**
Mean systolic BP^*^, mmHg (SD)	147 (12)	144 (10)
Mean diastolic BP^*^, mmHg (SD)	87 (11)	90 (8)
Mean number of antihypertensive drugs, *n* (SD) [min; max]	1.9 (0.8) [1; 4]	1.8 (0.9) [1; 4]
**Antihypertensive drugs used**, ***n*** **(%)**		
Diuretics	21 (46%)	18 (42%)
ACE inhibitors	13 (28%)	14 (33%)
Ang II receptor blockers	28 (61%)	21 (49%)
Calcium antagonists	13 (28%)	14 (33%)
Beta-blockers	10 (22%)	10 (23%)
Other	0 (0%)	0 (0%)
**Number of antihypertensive drugs**, ***n*** **(%)**		
0	0 (0%)	0 (0%)
1	14 (30%)	20 (47%)
2	23 (50%)	13 (30%)
≥3	9 (20%)	10 (23%)

*UC, usual care; TBC, team-based care; SD, standard deviation; ACE, angiotensin converting enzyme; Ang, angiotensin*.

* *The BP reported at baseline was mean daytime ABPM. If ABPM was not available at baseline, BP was based on automated office BP measurements and computed as the average of the last 3 out of 6 measurements with the patient resting alone quietly (24)*.

Table 3 shows that systolic and diastolic BP decreased in both groups during follow-up. At 6 months, the ITT between-groups difference in daytime systolic/diastolic ABPM was−3/+2 mmHg [95% confidence interval (CI):−10 to +4/-1 to +6; *p* = 0.45/0.20] and the systolic/diastolic control was 42%/48% in the TBC group and 39%/52% in the UC group (*p* = 0.63/0.45), respectively. At 12 months, the ITT between-groups difference in daytime ABPM was−7/-2 mmHg [95% CI:−13 to−2/-5 to +2; *p* = 0.01/0.42]; the systolic/diastolic control was 44%/53% in TBC group and 26%/48%% in UC group (*p* = 0.07/0.33), respectively. Upon adjustment for covariates in a set of linear regression models of growing complexity, the between-groups difference in systolic and diastolic ABP at 6- and 12-months of follow-up was maintained allowing to exclude important biasing effect of imbalance between groups (see Supplementary Tables S1, S2 in supplementary material). Of note, upon adjustment for baseline number of antihypertensive treatments and baseline BP, the ITT between-groups difference in daytime systolic/diastolic ABPM was maintained at 12 months of follow-up (i.e.,−5/-3 mmHg [95% CI:−10 to−1/-6 to 0; *p* = 0.02/0.09]. Finally, per-protocol analyses yielded similar results (see Supplementary Table S2 in supplementary material).

**Table 3:** Daytime systolic/diastolic ambulatory blood pressure monitoring (ABPM) and control at 6- and 12-month of follow-up.

	**6-month of follow-up**				**12-month of follow-up**			
	**UC**	**TBC**	^*^Δ [95% CI]	^*^p-value	**UC**	**TBC**	^*^Δ [95% CI]	^*^p-value
	***n* = 42**	***n* = 39**			***n* = 41**	***n* = 35**		
Mean systolic ABPM, mmHg (SD)	140 (17)	137 (17)	−3 [-10 to +4]	0.45	141 (14)	134 (14)	−7 [-13 to−2]	0.01
Mean diastolic ABPM, mmHg (SD)	83 (8)	85 (9)	2 [-1 to +6]	0.20	84 (10)	81 (8)	−2 [-5 to +2]	0.42
Systolic ABPM <135 mmHg, *n* (%)	39%	42%		0.63	26%	44%		0.07
Diastolic ABPM <85 mmHg, *n* (%)	52%	48%		0.45	48%	53%		0.33

* *The mean between group difference (Δ) and related statistical significance are computed following the intention-to-treat principle (ITT)*.

*UC, usual care; TBC, team-based care; SD, standard deviation; CI, confidence interval*.

Table 4 summarizes antihypertensive drug use during follow-up. In both groups, the mean number of antihypertensive drugs taken by TBC and UC patients slightly increased during follow-up. There was no difference between groups in the mean number of antihypertensive drugs at 6- and at 12-month of follow-up. The type of antihypertensive treatment taken did not change substantially during follow-up and between groups. However, patients in the TBC group tended to have experienced more frequently a switch to another class of drug as well as an increase of dosage or number of drugs. When all types of drug changes were considered, the mean number of changes per patient was greater in the TBC group compared to the UC group, at 6- and 12-months of follow-up.

**Table 4:** Antihypertensive drugs at 6- and 12-month of follow-up.

	**6-month of follow-up**			**12-month of follow-up**		
	**UC**	**TBC**	***p*-value**	**UC**	**TBC**	***p*-value**
	***n* = 42**	***n* = 39**		***n* = 41**	***n* = 35**	
Number of antihypertensive drugs, mean (SD)	2.1 (0.9)	2.1 (1.0)	0.93	2.1 (0.9)	2.3 (0.9)	0.43
**Number of antihypertensive drugs**, ***n*** **(%)**			0.33			0.62
0	0 (0%)	1 (3%)		0 (0%)	0 (0%)	
1	12 (29%)	13 (33%)		10 (24%)	7 (20%)	
2	19 (45%)	11 (28%)		20 (49%)	15 (43%)	
≥ 3	11 (26%)	14 (36%)		11 (27%)	13 (37%)	
**Class of antihypertensive drugs used**, ***n*** **(%)**			0.68			0.57
Diuretics	21 (52%)	16 (41%)		22 (54%)	19 (54%)	
ACE inhibitors	12 (29%)	13 (33%)		11 (27%)	14 (40%)	
Ang II receptor blockers	27 (64%)	22 (56%)		27 (66%)	22 (63%)	
Calcium antagonists	13 (31%)	17 (44%)		14 (34%)	12 (34%)	
Beta-blockers	9 (21%)	12 (31%)		8 (20%)	12 (34%)	
Other	1 (3%)	0 (0%)		2 (5%)	0 (0%)	
**Antihypertensive-drug modifications**						
Drug changes (change to another class of drug), *n* (%)	2 (5%)	6 (15%)	0.11	4 (10%)	9 (26%)	0.07
Drug intensifications (increase of dosage or number of drugs), *n* (%)	14 (33%)	19 (49%)	0.16	17 (41%)	21 (60%)	0.11
Drug reductions (decrease of dosage or number of drugs), *n* (%)	4 (10%)	7 (18%)	0.27	6 (15%)	4 (11%)	0.68
Any drug modification, *n* (%)	17 (40%)	21 (54%)	0.23	24 (59%)	24 (69%)	0.37
Mean number of drug modifications/patient (min–max)	0.6 (0–3)	1.1 (0–4)	0.04	0.9 (0–3)	1.3 (0–4)	0.06

*UC, usual care; TBC, team-based care; SD, standard deviation; ACE, angiotensin converting enzyme; Ang, angiotensin*.

### Difficulties to Medications and Lifestyle and Recommendations During Follow-Up

Among TBC patients, a total of 174 difficulties in 43 patients related to medications and lifestyle (such as lack of knowledge, beliefs, difficulties to integrate drugs in daily life) were identified (132/174 (75%) by the nurses and 42/174 (24%) by the pharmacists) during the first 6-months of follow-up. Nurses reported 83 (47%) issues related to physical activity and 49 (28%) to dietary and lifestyle habits (e.g., lack of motivation or lack of time to implement the change). Pharmacists reported mostly on medication adherence (15/42; 36% e.g., too many drugs to take daily, omission to take drugs or difficulties to integrate drugs in the daily activities of the patients). Another barrier frequently reported by pharmacists (14/42; 34%) was lack of knowledge concerning hypertension. During the same period, nurses made 164 recommendations related to dietary and lifestyle habits (most often to reduce salt consumption and to increase daily physical activity). Pharmacists made 40 recommendations related to hypertension drug treatment (most often patient adherence counseling about how to improve adherence (e.g., weekly reminder, clock alarm) and education about hypertension treatment.

## Discussion

The TBC-HTA randomized controlled study was one of the first pragmatic attempt to evaluate the effect of a TBC intervention involving community pharmacists and nurses working in collaboration with physicians to improve long term ambulatory BP in a European primary care setting under real conditions, that is, designed accounting for local constraints, resources, and expertise. While there was not significant effect on BP at 6 months of follow-up, the TBC intervention can help decrease long-term systolic BP among uncontrolled hypertensive patients.

### Comparison With Other Studies

Our results are consistent with previous studies (9, 11) and systematic reviews (8) reporting that physician-pharmacist collaboration can improve BP management and control. Our results are also congruent with the much fewer studies (30, 31) with a long-term follow-up, that is, beyond 6 months. Our results are also in line with the finding of a recent systematic review with meta-analysis of more than 100 trials and 55,920 patients showing that the most effective BP-lowering strategies use multilevel and multicomponent approaches to improve hypertension control, often involving non-physician providers assessing patients, measuring BP, and titrating medications as needed (6). In this review, the effect on BP of TBC with physician titrating medication was a mean 6.2/2.5 mmHg decrease in systolic/diastolic BP, which is close to the effect size of 7/2 mmHg seen in our study at 12 months of follow-up.

As underlined, the TBC-HTA study was pragmatic notably because it used the resources available and, the intervention was conducted by healthcare professionals involved in the follow-up of patients in their local setting. These human and local resources were used to conduct the study and the intervention which was designed accounting for the existing expertise. The fact that numerous patients screened were not included does not mean that we have excluded “real-world” patients. This is largely due to the lack of structured and specific practitioner database used in the different healthcare setting involved, but not to highly selective criteria of inclusion. As a result, many participants initially screened did not have inclusion criteria and were not invited to participate. Moreover, to design our study, we did not refer a priori to a specific model of care. However, we can consider that implicitly the model of care used in our study was close to the Chronic Care Model (32), incorporating patients, providers, and system level intervention.

### Strengths and Limitations of the Study

The main strengths of our pragmatic randomized controlled study are its design, the long-term follow-up, and the close interprofessional collaboration between nurses, community pharmacists, and physicians to evaluate the effects of TBC intervention on BP control among uncontrolled treated hypertensive patients in primary care practices. The use of a 24-h ambulatory BP monitoring device to measure BP at 6- and 12-month according to a standard protocol was also an asset to evaluate more precisely the effect of TBC intervention on BP control compared to office BP measurement (33). One key strength is the evaluation of the effect of the intervention at 1 year of follow-up (i.e., 6 months after ending the intervention). This is of importance because few studies evaluating this type of team-based care intervention have had follow-ups of more than 6 months (34). Nevertheless, our study suggests that the TBC intervention had almost no effect at 6 months of follow-up, i.e., at the end of the period of active intervention. This could be partly due to regression to the mean, as mean BP decreased in both groups. Another explanation could be a Hawthorne effect (35) during the first 6-months of follow-up: participants in the control group might have improved, at least initially, their dietary and lifestyle habits, as well as their drug adherence, because they knew they were part of a study. Interestingly, a similar observation was made during the first 2 months of a 12-month randomized controlled study that we had conducted to evaluate the effect of a pharmacist intervention to improve adherence among hypertensive patients in primary care (9).

There are however several limitations to our study. One major limitation was the small sample size of the study. Consequently, we have a slightly underpowered study which limits the possibility to get a more confirmative result. This could be one of the reasons why we did not find a statistically significant between-groups difference in mean daytime systolic BP at 6 months of follow-up, despite a favorable trend in the TBC group. Nevertheless, despite the relatively small sample size, the beneficial effect on systolic BP was significantly superior that usual care at 12 months. As with many randomized controlled trials, recruitment of sufficient number of patients was challenging and we did not reach the planned target sample size despite an extension of study time (36). Lack of a structured practitioner database and constrained human and financial resources were barriers to rapidly assess the potential eligibility of patients and ease recruitment. Furthermore, we could not pay healthcare providers to recruit patients.

Another limitation is that we conducted this study in selected outpatient clinics that were interested in implementing a TBC interprofessional model in their practice. This may limit the generalizability of our results. Further studies are needed to evaluate the transferability of our findings to other regions and populations. The absence of effect on diastolic BP is also a weakness. Patients were also not blinded to the intervention. Another limitation is the assessment and monitoring of lifestyles based on subjective assessments of healthcare providers. Digital monitoring technology, e.g., using smartphone (37) would have been better for a continuous assessment and a stronger involvement of patients (38). More broadly, the use of digital tools could have improved the completeness and effectiveness of the intervention.

Finally, this type of study does not allow the identification of the effects of each component of the TBC: is it the nurse or the pharmacist's intervention that makes a difference? Do changes in patients' diet and lifestyle impact BP control or are only medication changes important? Patients in the TBC group had actually more frequent changes in drug treatment during the follow-up. This suggests that one effect of the intervention was to decrease prescribing physician inertia, and that is consistent with other studies having shown that the intervention of another healthcare professional in the relationship between a patient and the physician improves BP control primarily through a reduction in inertia rather than through other mechanisms (12, 39). The difference in the components of interprofessional interventions also explains the heterogeneity of the effect size in studies having assessed such interventions on BP control and highlight the importance to evaluating locally these types of complex interventions (8). Our study also underlines the complexities of conducting such team-based care approach in real care setting.

## Conclusion and Further Perspective

While there was not significant effect on BP at 6-months of follow-up, our study shows that a TBC intervention could improve long-term systolic BP control among hypertensive patients in real-life conditions and hence supports interprofessional collaboration between nurses, community pharmacists and physicians to improve BP management in clinical practice, in line with recent North American (19) and European guidelines (ESC/ESH) (20).

Moreover, a team-based care practice or integrated care with, e.g., the support of digital solutions (telemonitoring, home blood pressure monitoring, or electronic health record) may also help manage hypertension by facilitating the exchange of information among the different healthcare professionals and by strengthening patient empowerment (40).

In conclusion, further studies are however still needed to evaluate, at large scale, how to implement efficiently this TBC model, for example by economic analyses, integrating cost and time estimations to provide the TBC intervention (41). These studies may offer policymakers additional compelling arguments and open good perspectives for an extensive implementation of the TBC model.

## Data Availability Statement

The original contributions presented in the study are included in the article/Supplementary Material, further inquiries can be directed to the corresponding author/s.

## Ethics Statement

This multicentric study was approved by the lead Ethics Committee (CER-VD: Cantonal Ethics Committee of Vaud on Research involving humans, ref. number 449/13), and by the local Ethics Committee (CCER: Cantonal Research Ethics Committee of Geneva, ref. number 15/281). The patients/participants provided their written informed consent to participate in this study.

## Author Contributions

VS, GW, AC, and MB designed and planned the study and accounting for substantial suggestions of LC, PS, and GP and contributed to the development of the training workshop. BP and AC conducted data analyses. VS, GW, and AC directed all aspects of study design and implementation. GW, PS, and MB fostered the clinic participation. VS, AC, and BP drafted the manuscript for publication and all co-authors made substantial contributions. All authors reviewed and approved the final manuscript.

## Funding

This study was supported by the Health Services Research funding program of the Gottfried and Julia Bangerter-Rhyner-Stiftung, the Swiss Academy of Medical Sciences (SAMS: www.samw.ch/en). This study received funding from the Swiss Society of Hypertension AstraZeneca (unrestricted grant-in-aid). The funder was not involved in the study design, collection, analysis, interpretation of data, the writing of this article or the decision to submit it for publication.

## Conflict of Interest

The authors declare that the research was conducted in the absence of any commercial or financial relationships that could be construed as a potential conflict of interest.

## Publisher's Note

All claims expressed in this article are solely those of the authors and do not necessarily represent those of their affiliated organizations, or those of the publisher, the editors and the reviewers. Any product that may be evaluated in this article, or claim that may be made by its manufacturer, is not guaranteed or endorsed by the publisher.
