# Supplementary material for: Team-Based Care for Improving Hypertension Management: A Pragmatic Randomized Controlled Trial
Source: Front Cardiovasc Med. 2021 Oct 25;8:760662. doi: 10.3389/fcvm.2021.760662 (PMC8572997; doi:10.3389/fcvm.2021.760662)
Supplement: Supplementary file 1 [file Data_Sheet_1.PDF]

Supplementary Material

SUPPLEMENTARY TABLE LEGENDS

Supplementary Table S1: Results of regression analyses using the intention-to-treat (ITT) principle.

Supplementary Table S2: Results of regression analyses using the per-protocol principle.

Table S1: Results of regression analyses using the intention-to-treat (ITT) principle. To assess the association of group allocation with systolic and diastolic daytime blood pressure (BP), respectively, three regression models of growing complexity were fitted with 1) no adjustment; 2) adjustments for age, sex, and centre; and 3) additional adjustments for the number of anti-hypertensive treatment at baseline and for baseline BP.

Follow-up	Regression model	Ajustement	Between-group difference in systolic BP (mmHg) (95% CI)	P-value	Between-group difference in diastolic BP (mmHg) (95% CI)	P-value
**6-months**	**Model 1**	No adjustment	-3 [-10 to 4]	0.45	2 [-1 to 6]	0.20
	**Model 2**	Adjustment for age, sex and center	-1 [-6 to 5]	0.87	3 [-1 to 6]	0.15
	**Model 3**	Adjustment for age, sex, center, antihypertensive treatment at baseline and BP at baseline	1 [-4 to 6]	0.71	2 [-1 to 5]	0.26
**12-months**	**Model 1**	No adjustment	-7 [-13 to -2]	0.01	-2 [-5 to 2]	0.42
	**Model 2**	Adjustment for age, sex and center	-6 [-11 to -1]	0.02	-2 [-5 to 1]	0.26
	**Model 3**	Adjustment for age, sex, center, antihypertensive treatment at baseline and BP at baseline	-5 [-10 to -1]	0.02	-3 [-6 to 0]	0.09

Table S2: Results of regression analyses using the per-protocol principle. To assess the association of group allocation with systolic and diastolic daytime blood pressure (BP), respectively, three regression models of growing complexity were fitted with 1) no adjustment; 2) adjustments for age, sex and centre and 3) additional adjustments for the number of anti-hypertensive treatment at baseline and for baseline BP.

**Follow-up**	**Regression model**	**Adjustment**	**Between-group difference in systolic BP (mmHg) (95% CI]**	**P-value**	**Between-group difference in diastolic BP (mmHg) (95% CI]**	**P-value**
**6-months**	**Model 1**	No adjustment	-3 [-10 to +5]	0.51	2 [-2 to 5]	0.33
	**Model 2**	Adjustment for age, sex and center	1 [-6 to +7]	0.87	2 [-2 to 6]	0.24
	**Model 3**	Adjustment for age, sex, center, antihypertensive treatment at baseline and BP at baseline	2 [-4 to +7]	0.50	2 [-2 to 5]	0.29
**12-months**	**Model 1**	No adjustment	-8 [-14 to -1]	0.02	-3 [-7 to 1]	0.16
	**Model 2**	Adjustment for age, sex and center	-6 [-11 to -1]	0.03	-3 [-7 to 0]	0.07
	**Model 3**	Adjustment for age, sex, center, antihypertensive treatment at baseline and BP at baseline	-5 [-10 to 0]	0.04	-4 [-7 to 0]	0.04
